# Platelet Reactivity and Response to Aspirin and Clopidogrel in Patients with Platelet Count Disorders

**DOI:** 10.1155/2021/6637799

**Published:** 2021-04-17

**Authors:** Wiktor Kuliczkowski, Ewa Żurawska-Płaksej, Maria Podolak-Dawidziak, Magdalena Cielecka-Prynda, Bożena Karolko, Jakub Dębski, Konrad Kaaz, Marcin Protasiewicz, Iwona Prajs, Andrzej Mysiak, Tomasz Wróbel, Lidia Usnarska-Zubkiewicz

**Affiliations:** ^1^Department of Cardiology, Wroclaw Medical University, Wrocław, Poland; ^2^Department of Pharmaceutical Biochemistry, Wroclaw Medical University, Wrocław, Poland; ^3^Department of Toxicology, Wroclaw Medical University, Wrocław, Poland; ^4^Department of Hematology, Blood Neoplasms, and Bone Marrow Transplantation, Wroclaw Medical University, Wrocław, Poland

## Abstract

**Background:**

Platelet reactivity and response to antiplatelet drugs, acetylsalicylic acid (ASA) and clopidogrel, in patients with thrombocytopenia and thrombocythemia can have a potentially important effect on the outcome. The effectiveness and safety of antiplatelet drugs in such patients has not been well examined. Measuring the effect of ASA and clopidogrel on platelets could help guide the therapy. Nevertheless, platelet response to antiplatelet drugs is not routinely measured in platelet count disorders and relevant evidence is scarce.

**Aims:**

The study aimed to measure platelet reactivity and response to ASA and clopidogrel in patients with platelet count disorders.

**Materials and Methods:**

This was a cross-sectional study of consecutive patients hospitalized in cardiology and hematology departments in the years 2018–2019. The study included patients with thrombocytopenia (PLT < 150 G/L) and thrombocythemia (PLT > 450 G/L) on ASA or dual antiplatelet therapy (DAPT; ASA plus clopidogrel). Controls included patients on antiplatelet drugs with normal platelet count. Platelet reactivity was measured in whole blood (Multiplate aggregometer, Roche, Switzerland) using arachidonic acid (AA), adenosine-5′-diphosphate (ADP), and thrombin receptor agonist peptide-6 (TRAP) as agonists. Platelet aggregation was expressed in arbitrary units (AU). AA-induced aggregation was used as a measure of response to ASA with a cut-off above 30 AU showing high on-treatment platelet reactivity to ASA (HTPR-A). ADP-induced aggregation measured response to clopidogrel with a cut-off above 48 AU for high on-treatment platelet reactivity to clopidogrel (HTPR-C). TRAP-induced aggregation measured baseline platelet reactivity not affected by oral antiplatelet drugs.

**Results:**

The study included 174 patients. There were 64 patients with thrombocytopenia, 30 patients with chronic thrombocythemia, and 80 controls. All patients were on 75 mg of ASA and 32% of them additionally on 75 mg of clopidogrel due to a history of recent coronary artery angioplasty. AA- and ADP-induced aggregation was comparable between thrombocytopenic patients and controls (median (IQR) 19 (7–28) vs. 23 (15–38) for AA AU and 32 (16–44) vs. 50 (32–71) for ADP AU, respectively), while it was significantly higher in thrombocythemic patients (median (IQR) 80 (79–118) for AA AU and 124 (89–139) for ADP AU). TRAP-induced aggregation showed significantly lowest aggregation in thrombocytopenic (median (IQR) 41 (34–60) for TRAP AU) and highest in thrombocythemic patients (median (IQR) 137 (120–180) for TRAP AU). HTPR-A was frequent in thrombocythemic patients in comparison with thrombocytopenic patients and controls (60% vs. 4% vs. 15%, respectively; *p* < 0.0002). HTPR-C was highly common in thrombocythemic patients and least common in thrombocytopenic ones in comparison with controls (80% vs. 8% vs. 40%, respectively; *p* < 0.001).

**Conclusion:**

Chronic thrombocytopenia does not significantly affect platelet reactivity and response to ASA and clopidogrel in comparison with controls. Thrombocytosis significantly increases platelet reactivity and attenuates response to both ASA and clopidogrel.

## 1. Introduction

Patients with platelet count disorders pose a challenge to cardiologists [1, 2]. The main reason is antiplatelet treatment which should be given to patients with coronary artery disease and concomitant chronic thrombocytopenia or thrombocytosis. Nevertheless, in such patients, there is an increased risk of bleeding and/or thrombotic events. Patients with thrombocytopenia were excluded from major modern antiplatelet drug trials and constituted less than 1% of participants in a trial on clopidogrel in myocardial infarction [2]. What the optimal dose of ASA and clopidogrel in such patients is and for how long and in what combination it should be administered is a matter of speculation only, although the percentage of patients with baseline thrombocytopenia can approach 6% in patients admitted for invasive cardiology procedures and it can independently influence the outcome [3]. The worse outcome which is noted in these patients can be explained by undiagnosed comorbidities which coexist with thrombocytopenia or patients'/doctors' reluctance to accept the standard antiplatelet therapy although newer data show that antiplatelet treatment in such population can be beneficial [4].

Thrombocytosis in cardiac patients is less frequent and less characterized [5]. There are some data on the laboratory monitoring of the effect of ASA in such patients but the results are conflicting [6]. What is known is the risk of stent thrombosis and restenosis, which is elevated [7].

Recent years have seen the rise and fall of the idea of laboratory monitoring of antiplatelet therapy. The knowledge of how ASA and P2Y_12_ receptor agonists block platelets could have provided information on how to safely conduct a therapy which can sometimes be years long. Nowadays, it is not advised routinely [8], although patients with platelet count disorders who need to be treated with antiplatelet drugs could benefit from the results of such tests [9].

Therefore, since data on antiplatelet treatment monitoring in low and high platelet counts is scarce, we consider further research in this field.

## 2. Aim

The study aimed to measure and compare platelet reactivity and response to antiplatelet drugs in patients with thrombocytopenia or thrombocytosis.

## 3. Materials and Methods

This was a cross-sectional study including consecutive patients with platelet count disorder admitted to the Department of Hematology, Blood Neoplasms and Bone Marrow Transplantation or the Department of Cardiology, Wroclaw Medical University, Poland. Thrombocytopenia was defined as a platelet count below 150 × 10^9^/L and thrombocytosis as a platelet count above 450 × 10^9^/L. We included patients with chronic platelet count disorders and excluded patients who developed platelet disorders (especially thrombocytopenia) during the index hospitalization. We aimed for patients with both concomitant platelet count disorders and coronary artery disease which resulted in the chronical use of ASA, and additionally clopidogrel in some of them, with a history of recent coronary angioplasty. The control group consisted of stable coronary artery disease patients on ASA, or ASA plus clopidogrel (dual antiplatelet treatment, DAPT), with normal blood platelet counts.

Platelet reactivity was measured with the use of whole blood impedance aggregometry (Multiplate, Roche, Switzerland). Aggregation agonists included arachidonic acid (AA) at a target concentration of 0.5 mM, adenosine diphosphate (ADP) at a target concentration of 6.4 *µ*M, and thrombin receptor agonist peptide (TRAP) at a target concentration of 32 *µ*M. The reagents were provided by the manufacturer of the aggregometer. Blood samples for aggregation measurement were always drawn an hour or two after morning drug administration. Aggregation was assessed within 2 h from blood sampling on hirudin as an anticoagulant, and results were expressed in arbitrary units (AU). Each aggregation measurement was performed twice, and the mean value was calculated. In the case of a 10% difference between measurements, the result was rejected and aggregation was repeated. AA was used as a measure of response to aspirin with a cut-off above 30 AU showing high on-treatment platelet reactivity to aspirin (HTPR-A). ADP-induced aggregation measured response to clopidogrel with a cut-off above 48 AU for high on-treatment platelet reactivity to clopidogrel (HTPR-C) [10]. TRAP-induced aggregation shows that baseline platelet reactivity is not affected by oral antiplatelet drugs. According to a recent report, platelet aggregation in thrombocytopenia was adjusted with the use of published formulas [11].

This study was approved by the Bioethics Committee of the Wroclaw Medical University in accordance with the Declaration of Helsinki. All patients provided written informed consent for the study.

### 3.1. Statistics

Data are presented as the mean and standard deviation or interquartile range. Normal distribution of data was examined with the Lilliefors test. Multiple comparisons in normal distribution data were performed with the ANOVA method, and the Kruskal–Wallis test was used for nonnormal distribution data. Multiple comparisons in nominal data were performed with the chi-squared test. The influence of covariates was checked through a linear model by the analysis of covariance (ANCOVA). *p* values were Bonferroni-adjusted for each test procedure. *p* values below 0.05 were considered statistically significant.

## 4. Results

The study included 174 patients (Table 1). There were 64 patients with chronic thrombocytopenia (51 with primary thrombocytopenia and 13 with unknown cause or during diagnosis), 30 patients with thrombocytosis (9 with polycythemia vera, 10 with essential thrombocytosis, and 11 with unknown cause or during diagnosis), and 80 patients with normal platelet count. All of them had coronary artery disease and were on 75 mg of aspirin, and 32% of them were additionally on 75 mg of clopidogrel due to recent coronary artery angioplasty.

**Figure 1:**
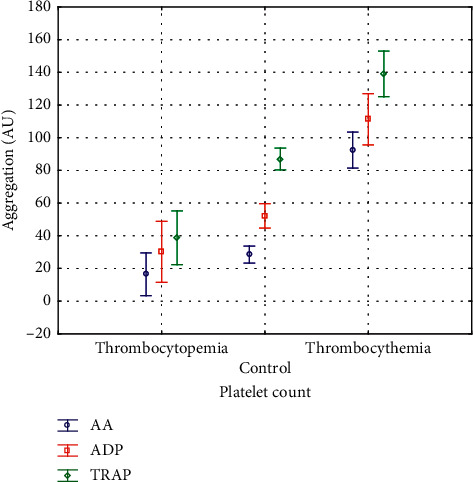
Platelet aggregation in patients with thrombocytopenia and thrombocythemia and controls. For significance of differences, refer to Table 2. AA: arachidonic acid-induced aggregation, ADP: adenosine diphosphate-induced aggregation, TRAP: thrombin receptor-activating peptide-induced aggregation, Box: mean, and Whiskers: 95% confidence intervals.

**Figure 2:**
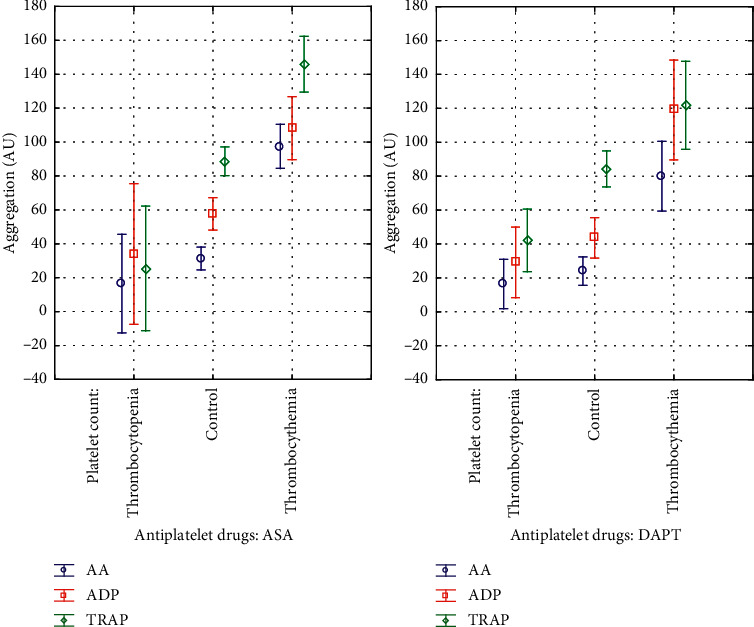
Platelet aggregation in patients with thrombocytopenia and thrombocythemia and controls according to the antiplatelet therapy used. For significance of differences, refer to Table 2. AA: arachidonic acid-induced aggregation, ADP: adenosine diphosphate-induced aggregation, TRAP: thrombin receptor-activating peptide-induced aggregation, ASA: acetylsalicylic acid, DAPT: dual antiplatelet therapy, Box: mean, and Whiskers: 95% confidence intervals.

There are two main findings of our study. First, platelet response to ASA and clopidogrel is comparable between chronically thrombocytopenic patients and controls and second, platelet response to ASA and clopidogrel is significantly attenuated in thrombocythemic ones. Specifically, AA- and ADP-induced aggregations were comparable between thrombocytopenic patients and controls and significantly increased in thrombocythemic patients when analyzed as the whole group as well as according to ASA or DAPT use (Table 2, Figures 1 and 2). HTPR-A was more frequent in thrombocythemic patients (60%; *p* < 0.0002) with no significant difference between thrombocytopenia (4%) and controls (15%). HTPR-C frequency showed a significant gradient with the lowest frequency in thrombocytopenia (8%) followed by controls (40%) and highest in thrombocytosis (80%) (*p* < 0.001).

As for baseline platelet reactivity, measured with TRAP-induced aggregation and not influenced by ASA and clopidogrel, there were significantly lowest values of aggregation in thrombocytopenic patients, higher in controls and highest in thrombocythemic patients (Table 2, Figures 1 and 2).

Importantly, according to the analysis of covariance (ANCOVA), there was no significant interaction between platelet reactivity to AA, ADP, and TRAP and the known cause of platelet count disorders (*p*=0.07), type of antiplatelet therapy used (*p*=0.27), and blood cell count parameters (WBC with *p*=0.53, RBC with *p*=0.68, and hematocrit with *p*=0.57).

## 5. Discussion

Results of our study show that in patients with chronic thrombocytopenia the level of platelet reactivity to ASA and DAPT measured with impedance aggregometry is comparable with controls. The effect of both drugs in patients with high platelet levels is significantly attenuated in comparison with controls and thrombocytopenic ones.

Current literature dealing with coronary patients and platelet count disorders focuses mainly on the clinical outcome and consists of case reports or a series of case reports [5, 12–15]. Laboratory measurement of platelet reactivity in such patients is challenging and underresearched [16]. The issue is even more complex when it comes to the measurement of platelet response to antiplatelet agents [17]. One of the reasons is that platelet count can influence platelet reactivity and response to ASA even within the normal range, with higher aggregation in higher platelet counts [18, 19]. In our study, we used whole blood aggregometry which is readily available and easy to perform. The problem with this method is that it seems to be sensitive to platelet count below 150 × 10^9^/L [20]. This limitation can be overcome after adjustment for low platelet count according to recently developed formulas [11]. In our study, we used this approach when analyzing the results of aggregation in thrombocytopenic patients, but even without this adjustment, we obtained comparable results (data not shown).

Our results are concordant to some extent with a recently developed model of thrombocytopenia where significant and positive linear associations were found between platelet count and platelet aggregation across all agonists used in the study, including, like in our study, TRAP and ADP [11]. The novelty of our data consists in the somewhat surprising result revealing that patients with thrombocytopenia on antiplatelet therapy show a laboratory effect of this therapy comparable to controls. To the best of our knowledge, this is the first observation of such an effect. We have to stress that in the study we included patients with chronic stable thrombocytopenia with only 5 patients with a platelet count below 50 × 10^9^/L. Above this cut-off value, it is believed to be safe to use ASA and even DAPT for a short time when it is necessary, although this is just an opinion of experts [2]. In this regard, our results reaffirm this opinion.

In patients with myeloproliferative disorders (thrombocytosis), multiple electrode aggregometry can be a valid method used for monitoring response to ASA and clopidogrel [21, 22]. A recent report showed that, in concordance with our study, in comparison with control patients, those with thrombocytosis have attenuated response to ASA when measured with whole blood aggregation and increased reactivity to TRAP [6].

Like in measuring platelet reactivity in thrombocytopenia, in thrombocytosis we can also encounter methodological limitations. It was shown in some studies that in thrombocytosis, WBC count or hematocrit can influence the response to whole blood aggregation [18, 21]. When adjusted to WBC count, increased platelet reactivity related to platelet level lost its significance [21]. In view of the above, we performed the ANCOVA analysis and did not confirm this association; neither did we find such association between platelet aggregation and RBC, hemoglobin, or hematocrit level.

In patients with essential thrombocytosis following a coronary intervention, HTPR-A was present in 26% and HTPR-C in 20% [14]. This percentage is lower than that in our study, which can be explained by the fact that different tests were used to measure it and patients were examined after cytoreductive treatment.

Our results can be reassuring for daily clinical practice; where at least from a laboratory point of view, giving a standard dose of ASA or clopidogrel to chronic thrombocytopenic patients (with platelet count mainly in the range 50–150 × 10^9^/L) will have a similar laboratory effect as in patients with normal platelet level. Our results can also prompt the decision to consider higher doses of antiplatelet agents in patients with platelet levels higher than normal, especially in those with a history of thrombotic events while already on ASA or clopidogrel, to avoid future events. In patients with thrombocytosis with confirmed laboratory HTPR to ASA or clopidogrel, one of the possible solutions would be doubling the dose of ASA and/or clopidogrel and splitting it into morning and evening administration. However, it should be stressed that such approaches are only speculative and need further studies on clinical, not laboratory, outcomes.

### 5.1. Study Limitation

Our study lacks clinical data on bleeding and thrombotic events. We also do not have data on all underlying diseases which provoked platelet count disorders, because some of the patients in our study were during the diagnostic process or did not have an established hematological diagnosis. Another issue is the sensitivity of Multiplate aggregometry to low platelet counts. As already mentioned, we used a recently published approach to overcome this issue [11], although it can still have some unrecognized effects on the results. This could have been overcome by flow cytometry or performing light transmittance aggregometry using samples diluted to maintain a constant platelet count.

Again, it has to be emphasized that overall, our results pertain to a laboratory setting, not a clinical one.

## 6. Conclusion

Chronic thrombocytopenia does not significantly affect platelet response to ASA and clopidogrel in comparison with controls. Thrombocytosis significantly increases platelet reactivity and attenuates the response to both ASA and clopidogrel.

## Figures and Tables

**Table 1:** Patients' characteristics.

Variable	Patients with thrombocytopenia (*n* = 64)	Patients with thrombocytosis (*n* = 30)	Controls (*n* = 80)	Statistical significance
	(1)	(2)	(3)	
Platelet count (G/L), mean ± SD (median, min-max)	73 ± 34 (79, 11–139)	683 ± 230 (567, 452–1136)	211 ± 62 (195, 150–371)	*p* < 0.000001 (1) vs. (2) vs. (3)
ASA, *n* (%)	64 (100)	30 (100)	80 (100)	NS
Clopidogrel, *n* (%)	15 (23)	9 (30)	31 (38)	NS
Age, years (mean ± SD)	68.2 ± 10.6	54.4 ± 24.6	61 ± 8.5	*p* < 0.01 (1) vs. (2), (1) vs. (3)
WBC (mean ± SD)	6 ± 4.3	8.3 ± 2	7 ± 2.8	NS
RBC (mean ± SD)	3.7 ± 0.8	4.2 ± 0.6	4.2 ± 0.7	*p* < 0.05 (1) vs. (3)
Hb (mean ± SD)	10.7 ± 2.2	12.8 ± 2.0	12.7 ± 2.3	*p* < 0.01 (1) vs. (2) (1) vs. (3)
Arterial hypertension, *n* (%)	21 (33)	12 (40)	37 (46)	NS
Diabetes mellitus, *n* (%)	11 (17)	6 (20)	20 (25)	NS
Kidney insufficiency, *n* (%)	9 (14)	5 (16)	10 (12)	NS
PCI history, *n* (%)	15 (23)	7 (23)	49 (61)	*p* < 0.05 (1) vs. (3) (2) vs. (3)
CABG history, *n* (%)	4 (6)	2 (7)	8 (10)	NS

ASA: acetylsalicylic acid, WBC: white blood cells, RBC: red blood cells, PCI: percutaneous coronary intervention, CABG: coronary artery bypass grafting, Hb: hemoglobin, SD: standard deviation, and NS: no statistical significance (*p* value above 0.05).

**Table 2:** Platelet reactivity presented as a median and 25%–75% interquartile range, IQR.

Aggregation agonist	Aggregation in patients with thrombocytopenia	Aggregation in patients with thrombocythemia	Aggregation in controls	Statistical significance
	(1)	(2)	(3)	
AA whole group (*n* = 174)	19 (7–28)	80 (79–118)	23 (15–38)	(2) vs. (1) *p*=0.001 (2) vs. (3) *p*=0.001
AA on ASA only (*n* = 119)	14 (5–29)	80 (79–118)	23 (17–38)	(2) vs. (1) *p*=0.004 (2) vs. (3) *p*=0.005
AA on DAPT only (*n* = 55)	19 (7–26)	80 (70–110)	20 (15–31)	(2) vs. (1) *p*=0.004 (2) vs. (3) *p*=0.005
ADP whole group (*n* = 174)	32 (16–44)	124 (89–139)	50 (32–71)	(2) vs. (1) *p*=0.005 (2) vs. (3) *p*=0.005
ADP on ASA only (*n* = 119)	36 (10–65)	136 (89–139)	52 (41–73)	(2) vs. (1) *p*=0.003 (2) vs. (3) *p*=0.004
ADP on DAPT only (*n* = 55)	30 (18–41)	119 (114–124)	36 (21–67)	(2) vs. (1) *p*=0.001 (2) vs. (3) *p*=0.001
TRAP whole group (*n* = 174)	41 (34–60)	137 (120–180)	90 (70–104)	(1) vs. (2) *p*=0.002 (2) vs. (3) *p*=0.002 (1) vs. (3) *p*=0.0002
TRAP on ASA only (*n* = 119)	36 (15–72)	137 (125–180)	86 (71–105)	(1) vs. (2) *p*=0.01 (2) vs. (3) *p*=0.01 (1) vs. (3) *p*=0.002
TRAP on DAPT only (*n* = 55)	42 (39–53)	129 (104–139)	93 (65–103)	(1) vs. (2) *p*=0.01 (2) vs. (3) *p*=0.01 (1) vs. (3) *p*=0.002

AU: arbitrary units, AA: arachidonic acid, ADP: adenosine diphosphate, TRAP: thrombin receptor-activating peptide, ASA–acetylsalicylic acid, DAPT: dual antiplatelet therapy, and IQR: 25%–75% interquartile range.

## Data Availability

The data used to support this study are available from the corresponding author upon reasonable request (wiktor.kuliczkowski@umed.wroc.pl).
